# Reactive plasmacytosis mimicking multiple myeloma associated with SFTS virus infection: a report of two cases and literature review

**DOI:** 10.1186/s12879-018-3431-z

**Published:** 2018-10-22

**Authors:** Jinjing Zhang, Xiaojing Yan, Yan Li, Ran Gao, Pingping Wang, Wenbin Mo

**Affiliations:** Department of Hematology, The First Affiliated Hospital of China Medical University, Shenyang, 110001 Liaoning China

**Keywords:** Severe fever with thrombocytopenia syndrome, SFTS, Reactive plasmacytosis, Multiple myeloma

## Abstract

**Background:**

Severe fever with thrombocytopenia syndrome (SFTS) is an emerging infectious disease caused by a novel bunyavirus named SFTS virus (SFTSV), which is classified into the genus *Phlebovirus* and family *Phenuiviridae*. Reactive plasmacytosis mimicking multiple myeloma is a very rare condition in association with SFTS. Here, we describe two SFTS cases who presented with hyperimmunoglobulinemia, as well as extensive bone marrow and peripheral blood plasmacytosis, which mimicked multiple myeloma (MM).

**Case presentation:**

We report two cases who presented with fever and blood routine abnormity which were conformed as SFTS eventually. They were performed bone marrow aspiration and were admitted to the department of hematology with a preliminary diagnosis of MM. They all had hyperimmunoglobulinemia, extensive bone marrow and peripheral blood plasma cells, prolonged activated partial thromboplastin time (APTT), elevated hepatic enzyme. The two patients recovered with treatment of doxycycline, human immunoglobulins, plasma transfusion, and other supporting treatments. But case 1 occurred lymphoma 8 months later and died.

**Conclusion:**

SFTS might be one of differential diagnosis of MM in certain endemic area. We also conclude that SFTSV is a pantropic virus that could injure most tissues and cells of the human body.

## Background

Severe fever with thrombocytopenia syndrome (SFTS) is an emerging infectious disease with a constellation of clinical signs and symptoms, including fever, gastrointestinal symptoms, hemorrhagic tendency, thrombocytopenia, leukocytopenia, elevated serum enzyme levels, and regional lymphadenopathy [1]. The etiological pathogen, severe fever with thrombocytopenia syndrome virus (SFTSV), was first identified by Chinese Center for Disease Control and Prevention (China CDC) in 2010 [2]. Ticks are considered potential transmission vectors of SFTS, however, there have been several reports of human-to-human transmission [3–5]. Although SFTS has multiple manifestations, reactive plasmacytosis is an extremely rare condition in association with SFTS. Here we describe two patients with SFTS who presented with reactive plasmacytosis, mimicking multiple myeloma (MM). In addition, we discuss the clinical and laboratory characteristics of the SFTS cases. Also, the clinical data of 4 Asian patients with SFTS associated reactive plasmacytosis is summarized.

## Cases presentation

### Case 1

**Fig. 1:**
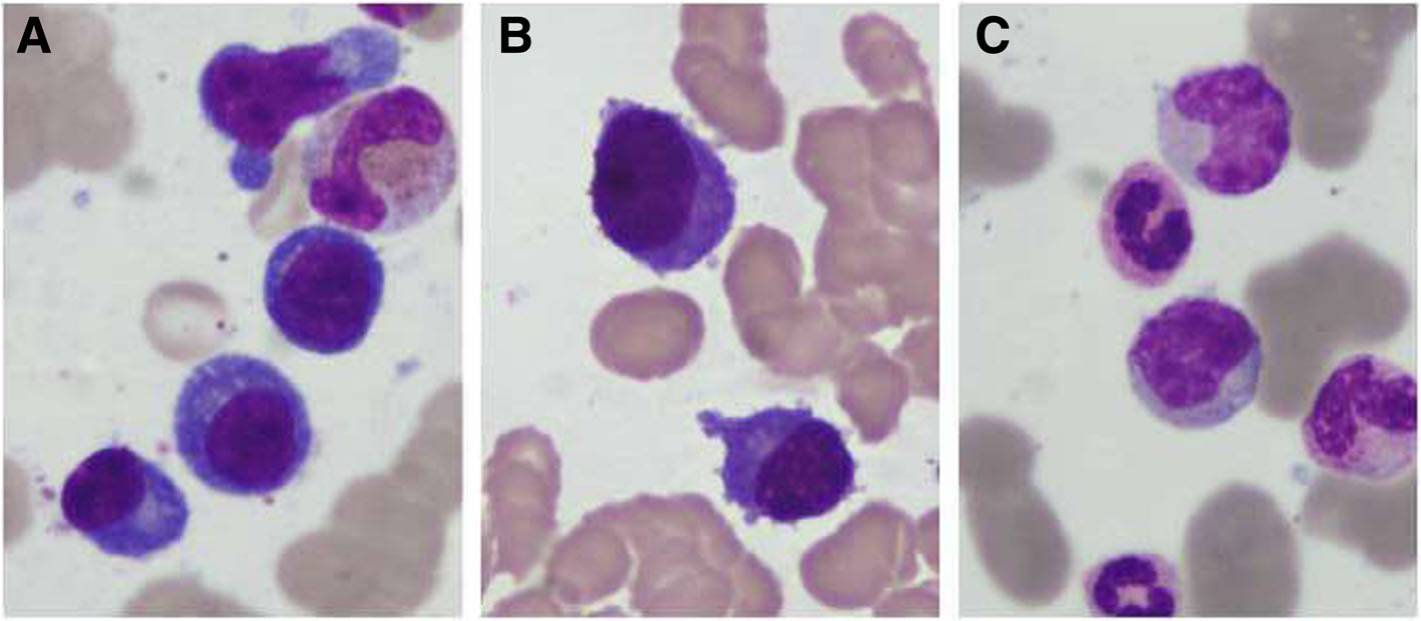
Representative images of bone marrow cytology(Wright and Giemsa stain) showing plasmacytosis. **a** For case 1, (**b** and **c**) for case 2 at different time points

**Fig. 2:**
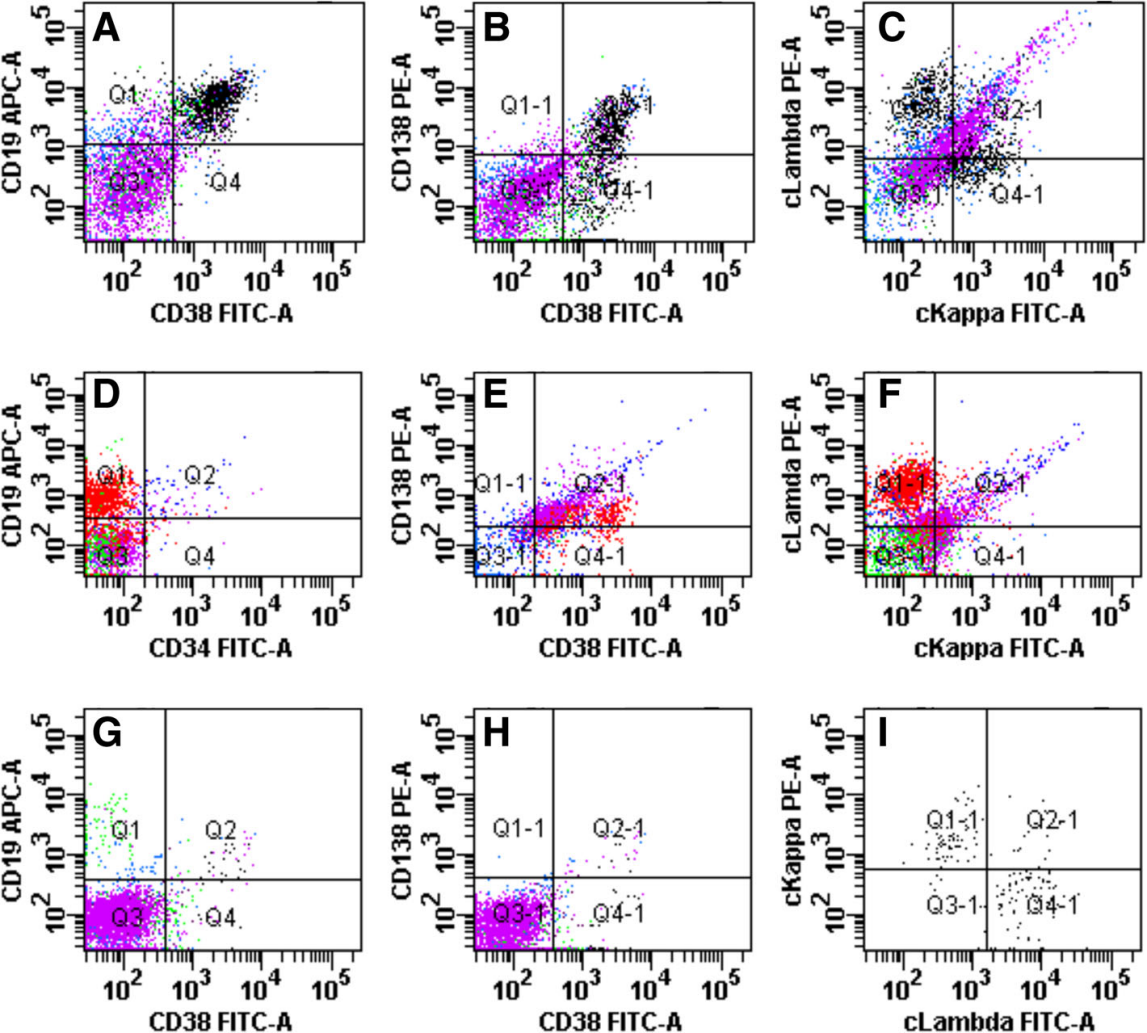
Representative scatter plots of bone marrow flow cytometric immunophenotyping. (**a**-**c**) For case 1, the incresed plasma cells were polyclonal (CD19 + CD38 + CD138 + cKappa+cLambda+) (**d**-**f**) for case 2, the incresed plasma cells were abnormal monoclonal (CD19 + CD38 + CD138 + cKappa-cLambda+) (**g**-**i**) for case 2, the incresed plasma cells disappear and were polyclonal (CD19 + CD38 + CD138 + cKappa+cLambda+)

A 63 year-old male farmer with a history of a tick bite presented with a sudden onset of fever on May 28, 2014, with a temperature of 38.6 °C, accompanied by rash, nausea, anorexia, fatigue, enlarged lymph node, and general body aches. He presented to the emergency department of The First Affiliated Hospital of China Medical University (CMU) on June 4. Routine blood tests showed leukocytosis with peripheral blood plasmacytosis (white blood cell [WBC] count, 24.46 × 10^9^/L; proplasmacytes, 5%; mature plasma cells, 18%) and thrombocytopenia (platelets[PLT], 75 × 10^9^/L). Cytological examination of the bone marrow demonstrated plasmacytosis. With a preliminary diagnosis of MM, he was admitted to the Department of Hematology of CMU on June 6. Physical examination revealed palpable swollen lymph nodes in the submandibular and bilateral axillary regions, accompanied with dispersed red papules on chest and abdomen. Laboratory tests upon admission showed thrombocytopenia, increased alkaline phosphatase (ALP) and lactate dehydrogenase (LDH) levels, prolonged prothrombin time (PT) and activated partial thromboplastin time (APTT). Immunofixation by electrophoresis revealed a polyclonal pattern with increased amounts of immunoglobulin (IgA, IgG, and IgM) and immunoglobulin light chains. Proteinuria was observed but Bence Jones proteinuria was negative. Because of abnormal bone marrow cytology, X-ray of skull, thoracic and lumbar vertebrae, and pelvis was performed, with normal findings. ELISA and RT-PCR were performed to detect SFTSV-specific IgM/IgG and SFTSV RNA as previously described [2, 6]. And the IgM antibody and RNA to SFTSV were positive. Bone marrow cytology showed plasmacytosis, with plasma cells accounting for 29.2% of all nucleated cells, including proplasmacytes (6.8%) and mature plasma cells (22.4%) (Fig. 1). However, flow cytometric immunophenotyping (FCI) of bone marrow revealed that the increased plasma cells were not monoclonal (Fig. 2). The patient was administered doxycycline, human immunoglobulins, plasma transfusion and other supporting treatments, and was improved. Finally, a diagnosis of reactive plasmacytosis associated with SFTS was reached. The clinical and laboratory findings are summarized in Table 1.

**Table 1:** Clinical findings and laboratory data of the two patients with SFTS who presented with reactive plasmacytosis

Gender/age,y	M/63	F/42
Occupation	farmer	farmer
Onset to admission, d	8	7
Tick bite history	+	+
Fever	+	+
Gastrointestinal symptoms^a^	+	+
Lymphadenopathy	+	+
Rash	+	+
Fatigue	+	_
hemorrhage^b^	_	+
Apathy	_	+
WBC (3.5–9.5^a^10~ 9/L)	24.46	2.58
RBC(M: 4.3–5.8^a^10~ 12/L, F: 3.8–5.1^a^10~ 12/L)	3.86	4.03
PLT(125–350^a^10~ 9/L)	75	25
PT(11.0–13.7 s)	16.9	17.5
Fg(2.00–4.00 g/L)	2.9	1.82
APTT(31.5–43.5 s)	68.3	90.0
Proteinuria	1+	microscale
AMY(28–100 U/L)	30	230
LPS(13–60 U/L)	27	194
ALT(13–69 U/L)	37	727
AST(15–46 U/L)	28	2940
ALP(38–126 U/L)	244	120
LDH(135–225 U/L)	794(313–618 U/L)	2690
CK(39–308 U/L)	21	1086
Immunofixation electrophoresis	polyclonal IgG	polyclonal IgG
IgA(0.82–4.53 g/L)	2.64	2.43
IgG(7.51–15.6 g/L)	17.2	28.5
IgM(0.46–3.04 g/L)	28.1	0.76
Antibody IgM, RNA of SFTSV	+	+
Bone marrow cytology	Plasma cells accounting for 29.2%: naïve (6.8%) and mature (22.4%) ones.	(2014-7-18) Plasma cells accounting for 50.4%: naïve (39.6%) and mature (10.8%); (2014-7-31) normal mature plasma cells accounting for 2.2%, no naive plasma cells.
Flow cytometric immunophenotyping(FCI)	Plasma cells of normal phenotype accounting for 20%, mainly expressing CD38, CD 138, CD19; partly expressing CD 200, cKappa, and cLambda, not expressed CD20, Kappa, Lambda, CD25, CD35, CD22, FMC7, CD103, CD10, CD5, IgM, CD23, CD117, and CD56.	(2014-7-18) Plasma cells of abnormal phenotype accounting for 44.7%, mainly expressing CD38, CD 138, CD19, and cLambda; not expressing CD7, CD117, CD33, CD10, CD34, CD28, CD56, CD25, CD11c, CD5, FMC7, CD22, TdT, CD200, CD20, Kappa, Lambda, and cKappa. (2014-7-31) Plasma cells of normal phenotype accounting for 1.1%, mainly expressing CD38, CD138, CD19, partly expressing cKappa and cLambda.
outcome	Recoverd	Recoverd^c^

^a^ Nausea, vomiting, anorexia, or abdominal discomfort

^b^ Multiple skin petechiae or ecchymosis

^c^ This patient was diagnosed with angioimmunoblastic T-cell lymphoma 8 months later and died eventually

### Case 2

A 42 year-old female farmer who had a sudden onset of fever on July 7, 2014, with a temperature of 39.2 °C, accompanied by rash, nausea, and multiple skin petechiae or ecchymosis. She visited a local hospital on July 14, 2014. Laboratory examination indicated pancytopenia, increased aspartate aminotransferase (AST) and alanine aminotransferase (ALT) levels, and normal coagulation index. Three days later, no clinical improvement was observed, and she was admitted to the Hematology Department of The First Affiliated Hospital of CMU. Laboratory tests found prolonged APTT and decreased fibrinogen (Fg). Serum enzymes, including creatine kinase (CK), LDH, and AST, were also assessed. Serum IgM and IgG antibodies and RNA to SFTSV in patient samples were detected by ELISA and RT-PCR respectively which were performed by Liaoning CDC as previously described [2, 6]. The patient was suspected with SFTSV infection, and sent to the Department of Infectious Disease of our hospital on July 22. Meanwhile, serum amylase and lipase levels were obviously elevated, with no abdomen pain; however, the patient showed apathy, with suspicious neck stiffness. Certain amounts of abnormal plasma cells were found in both BM (proplasmacytes, 39.6%; mature plasma cells, 10.8%) (Fig. 1) and peripheral blood (proplasmacytes, 10%; mature plasma cells 36%). In addition, about 44.7% suspicious abnormal monoclonal plasma cells were determined by FCI on July 23 (Fig. 2). Thus, malignant plasma cell disease could not be excluded. The immunoglobulins (IgA, IgG, and IgM) and their light chains were polyclonal as tested by Immunofixation. Here also, proteinuria was positive but Bence Jones proteinuria was negative. On July 26, gastrointestinal symptoms such as nausea, vomiting and abdominal discomfort began to appear; abdominal enhanced computed tomography was performed, and indicated pancreatic edema and multiple swollen lymph nodes in the left inguinal and inner thigh regions. Doxycycline, human immunoglobulins, somatostain, plasma transfusion, and the other supportive medications were administered to the patient. On July 31, the IgM antibodies and nucleic acid of SFTSV were detected, with her clinical symptoms improved. In the meantime, abnormal plasma cells in the bone marrow and blood were absent at reexamination of bone marrow cell morphology (Fig. 1) and FCI (Fig. 2). Eventually, a diagnosis of reactive plasmacytosis in association with SFTS was reached. These clinical findings are summarized in Table 1.

## Discussion and conclusion

In September 2006, the first SFTS case occurred in Dingyuan country, Chuzhou, Anhui province [5]. Since then, sporadic cases mainly manifested with fever and thrombocytopenia have been reported one after another in several provinces of China. In 2010, the responsible pathogen isolated from patients was named SFTSV, a novel bunyavirus. From 2010 to October 2016, more than 7000 SFTS cases have been diagnosed in China, with the average mortality of 5.3% right now, while the mortality rates of SFTS ranges from 12 to 30% at the early days of people’s recognition for this type of disease [1, 7]. Because of nonspecific clinical features of SFTS ranging from head to toe, some patients with SFTS are misdiagnosed with other diseases such as common fever, gastrointestinal disease, human granulocytic anaplasmosis, hemorrhagic fever with renal syndrome, and leptospirosis [1]. Based on 743 confirmed cases in previous studies, the clinical characteristics of SFTS patients are summarized in Table 2 [1, 6, 8–12]. The main disease symptoms include fever (95.3%), body sores (81.1%), anorexia (75.4%), fatigue (77.7%), nausea (65.8%) and vomiting (51.5%). Other common clinical features include dizziness (47.0%), malaise (46.4%), chill (46.2%), diarrhea (44.8%), headache (43.9%), lymphadenopathy (42.8%), and cough (42.8%). In addition, joint pain and petechiae are observed in 34.1% and 23.3% patients, respectively. Furthermore, some cases present with nervous system symptoms, respiratory symptoms, and liver and spleen enlargement, respectively.

**Table 2:** Clinical symptoms of patients with severe fever with thrombocytopenia syndrome [1, 6, 8–12]

Symptoms or signs	Patients with SFTS(n)	Patients with symptoms or signs(n)	Percent(%)
Fever	743	708	95.3
Anorexia	574	433	75.4
Fatigue	574	446	77.7
Nausea	743	489	65.8
Vomiting	743	383	51.5
Diarrhea	743	333	44.8
Abdominal pain or tenderness	446	127	28.5
Headache	743	326	43.9
Body sores	238	193	81.1
Lymphadenopathy	731	313	42.8
Cough	603	258	42.8
Chill	662	306	46.2
Dizziness	423	199	47.0
Malaise	69	32	46.4
Muscular tremor	69	21	30.4
Petechiae	477	111	23.3
Arthralgia/joint pain	369	126	34.1
Throat congestion	196	44	22.4
Hematuria	115	22	19.1
Apathy	297	65	21.9
Confusion	81	18	22.2
Coma	297	36	12.1
Lethargy	228	37	16.2
Convulsion	228	27	11.8
Dyspnea	284	51	18.0
Skin rash	238	19	8.0
Chest pain	115	6	5.2
Hematemesis	343	33	9.6
Gingival bleeding	180	15	8.3
Hematoma on puncture sites	115	7	6.1
Conjunctival congestion	261	21	8.0
Hyperemia of face	115	9	7.8
Slurred speech	69	4	5.8
Hepatomegaly	115	7	6.1
Splenomegaly	115	5	4.3

Abnormal laboratory findings reported in previous studies [1, 6, 11] of SFTS cases revealed thrombocytopenia (96.0%), leukopenia (91.8%), elevated AST (95.4%), increased ALT (92.6%), elevated LDH (91.8%), low calcium (82.7%), proteinuria (78.0%), and prolonged APTT (77.2%). Serum levels of CK, creatine kinase MB fraction (CK-MB), AMY, LPS and creatinine (Cr) are high in patients with SFTS. Hematuria and fecal occult blood were also observed (Table 3). Taken together, these findings indicated that SFTSV is a pantropic virus that injures most tissues and cells; it can invade the hematological system, cardiac muscles, the liver, kidneys, and the coagulation system, inducing related symptoms and abnormal examination results [7, 13–15]. Therefore, early diagnosis and treatment is beneficial for patient survival.

**Table 3:** Laboratory findings in patients with severe fever with thrombocytopenia syndrome [1, 6, 11]

Laboratory findings	Patients with SFTS (n)	Patients with positive results (n)	Percent (%)
Thrombocytopenia	426	409	96.0
Leukopenia	427	392	91.8
Elevated alanine aminotransferase (ALT)	417	386	92.6
Elevated Aspartate aminotransferase (AST)	416	397	95.4
Elevated Lactate dehydrogenase (LDH)	404	371	91.8
Proteinuria	391	305	78.0
Low calcium	353	292	82.7
Prolonged activated partial-thromboplastin time (APTT)	127	98	77.2
Elevated Creatine kinase (CK)	402	269	66.9
Elevated Creatine kinase MB fraction (CK-MB)	144	93	64.6
Hematuria	46	27	58.7
Low sodium	353	157	44.5
Elevated serum amylase level (AMY)	48	18	37.5
Elevated serum lipase level (LPS)	48	7	14.6
Fecal occult blood	105	34	32.4
Elevated creatinine (Cr)	115	15	13.0

Herein, we described two special cases of SFTSV infection, with both patients exhibiting bone marrow plasmacytosis during the course of the disease. Although the cytology of bone marrow plasma cells mimicked multiple myeloma in case 1, BM cell immunophenotyping revealed benign, polyclonal plasma cell infiltration, compatible with reactive plasmacytosis. The Igs were polyclonal as assessed by immunofixation. In addition, the patient recovered after supportive therapy with no bone disease (lytic or osteopenic), anemia, renal insufficiency, or calcium elevation. However this patient presented with fever, lymphadenopathy and cough 8 months later and was diagnosed with angioimmunoblastic T-cell lymphoma (AITL) and died eventually. The question that whether there is relationship or not between AITL with SFTSV infection remain to be answered. In case 2, although abnormal plasma cells were found in both BM (including smear morphology and flow cytometry immunophenotyping) and peripheral blood (smear morphology) within 11 days of disease onset, diagnosis of plasma cell leukemia was ruled out because the increased Igs were polyclonal. Meanwhile, BM reexamination indicated normal cytology and immunophenotype within 24 days of disease onset, when the clinical manifestations and laboratory tests of the patient were improved, consistent with a reactive process. Reports of cytological changes, such as altered cell proliferation as well as histiocyte and phagocyte presence in the BM, in patients with SFTS are available [6, 16]. Moreover, a study found that bone marrow cell cytology in SFTS patients display similarity to that of healthy individuals [17]. However, reactive plasmacytosis mimicking multiple myeloma in the BM in association with SFTS is extremely rare. To our knowledge, reactive plasmacytosis characterized by transiently increased amounts of polyclonal plasma cells in the BM and peripheral blood, is an uncommon hematological event in SFTSV infection. Until now, only two other SFTS-associated reactive plasmacytosis cases have been reported in the Chinese and English literature [18, 19]. The common clinical features of these two cases include fever, thromobocytopenia and reactive plasmacytosis which are similar to our patients. All the four cases were diagnosed as SFTS by the positive results of SFTSV RNA detection. One report described a SFTS case with reactive plasmacytosis in both BM and peripheral blood without hyperimmunoglobulinemia [18]. The other report discussed a fatal SFTS case with reactive plasmacytosis in peripheral blood via flow cytometry without BM examination. The authors emphasized that clonality assessment of plasma cells was necessary to avoid misdiagnosis and delayed diagnosis in SFTS [19]. However, reactive plasmacytosis in SFTSV infection might be underestimated. There are two possible reasons for this. First, it is a transient event based on the reflection of immune reactions [20, 21]. Secondly, cell morphology in the bone marrow or blood smear is not assessed in most patients with SFTS because of the use of automated cell counters, which cannot identify plasma cells correctly [2].

Reactive plasmacytosis is a rare event found in a variety of diseases such as infectious diseases, tumors and autoimmune disorders [20]. Reactive plasmacytosis has been reported in several types of virus infections including Hepatitis A virus, Epstein-Barr virus, Dengue virus, Parvovirus B 19 [21–24]. In this report, we describe two unusual cases of SFTSV infection presenting with reactive plasmscytosis both in peripheral blood and bone marrow. These two patients were diagnosed with SFTS, but not complicated with tumors or autoimmune diseases, therefore we consider that reactive plasmacytosis could be accompanied with SFTSV infection just as the other types of virus infections reported previously. In the process of SFTSV infection, the virus and cytokines are detectable in blood, similar to what is seen in other virus infections [25]. Several studies reported that SFTSV infection could induce a cytokine storm, with increased levels of serum cytokines like IL-6, IL-10, MCP-1, G-CSF and IP-10, which might contribute to disease severity and outcome [26–28]. Among them, IL-6 does not only participate in the differentiation of B cells into plasma cells, but also affects the generation of plasma cells as shown in knockout mice [29, 30].

Furthermore, IL-6 plays a central role in the proliferation, differentiation, survival and immunoglobulin secretion in plasmablasts [31]. Therefore, we propose that excessive IL-6 production may be a potential explanation for plasmacytosis in SFTS patients. Further studies exploring the mechanisms of human SFTSV infection are warranted, to determine the exact role of plasma cells in SFTS pathogenesis and expand our knowledge of SFTSV infection.

Currently, several methods are available for differentiating polyclonal plasma cells from monoclonal plasma cells, including cell morphology, serum protein electrophoresis, immunofixation by electrophoresis, and flow cytometry immunophenotyping. Recently, the widespread use of FCI makes it more simple, efficient and accurate to distinguish benign plasma cells from malignant ones. Yet, a deviation may still occur, if FCI signals are solely used for diagnosis. Therefore, it is essential to take into consideration various auxiliary examination results, when seeking a diagnosis.

In summary, although this report described a particular phenomenon that occurs in SFTSV infection, the mechanism and potential role in SFTSV in reactive plasmacytosis remain unknown. Further research should be carried out to address these questions. Interestingly, it is studied that T cell proliferation, activation and apoptosis occoured in the SFTSV infection recently [32]. In addition, it is critical to make an early and correct diagnosis of SFTS, based on detailed epidemiological data such as onset season, occupation, residential address, working environment, and tick bite history, as well as a comprehensive analysis of clinical characteristics and laboratory findings. In the future, further education of physicians should be carried out in order to avoid misdiagnosis of SFTS and expand the knowledge of SFTSV infection. The clinicians should be aware that SFTS virus infection can be asooiciated with hematologic change such as reactive plasmacytosis.

## Abbreviations

ALP: Alkaline phosphatase
ALT: Alanine aminotransferase
APTT: Activated partial thromboplastin time
AST: Aspartate aminotransferase
CK: Creatine kinase
CK-MB: Creatine kinase MB fraction
Cr: Creatinine
FCI: Flow cytometric immunophenotyping
Fg: Fibrinogen
LDH: Lactate dehydrogenase
MM: Multiple myeloma
PT: Prothrombin time
SFTS: Severe fever with thrombocytopenia syndrome
SFTSV: SFTS virus
WBC: White blood cell
